# The Late General Myer—The Signal Service

**Published:** 1880-12

**Authors:** 


### THE LATE GENERAL ALBERT J. MYER—THE SIGNAL SERVICE.

Among the many notices which have been written of the late lamented chief of the Signal Service, we have read none which states correctly the particulars of the early life of General Myer, or the curious circumstances which led to the invention of the army signals, and to the whole system which has rendered its inventor famous.

General Myer and the editor of this journal were fellow students, room mates and class mates at Hobart College, which institution General Myer entered at the age of fourteen. Though the youngest member of his class, he was easily at the head in all branches of learning. We have seen one notice of the General in which his career is pronounced as being more remarkable for the reason that his early training was defective. This is an error. His early training was exceptionally careful, and few men of his age have been graduated with so high a standing. After leaving college he was a fellow student with us in the office of Dr. Frank H. Hamilton, the distinguished surgeon, then of Buffalo, now residing in New York. We were neither of us rich, and to increase our incomes we procured employment as telegraphers in one of the telegraph offices at Buffalo, upon a line which was under the control of a relative of the writer. Gen. Myer became an expert operator. It was in this office and in practicing the Morse alphabet that the idea first occurred to him that the telegraphic signs could be made useful in framing a sign language for deaf mutes. He thought much of the matter, and was constantly discussing it with his fellow operators. At our graduation at the Buffalo Medical College he presented a thesis entitled "Suggestions for a new Sign Language for Deaf Mutes," which attracted greatly the attention of the faculty and of the public, and was subsequently printed. In this thesis his whole system of sign language and signals, destined in the near future to become famous throughout the world, was foreshadowed. General Myer entered the army as an assistant surgeon, and the writer chose the practice of medicine, and we were separated. Soon afterward the civil war broke out, and General Myer, seeing the pressing need of some efficient system of army signals, was not long in devising the one which has made him famous. It was only the extension and development of the system which he had devised when he was a boy operator in the telegraph office at Buffalo. The rest is known to all the world, how he became famous as the inventor of a system which all the civilized world has adopted, and how in his very prime he was suddenly called away. The world has lost in him one of its really great men, and the writer a life-long and devoted friend.

---

A Touching Story.—One rarely meets a bit of more touching romance than is found in the following story, that comes from Wales: "Years ago some Welsh miners, in exploring an old pit that had long been closed, found the body of a young man dressed in a fashion long out of date. The peculiar action of the air of the mine had been such as to preserve the body so perfectly that it appeared asleep rather than dead. The miners were puzzled at this circumstance; no one in the district had been missed within their remembrance, and at last it was resolved to bring the oldest inhabitant—an old lady long past her eightieth year, who had lived single in the village the whole of her life. On being brought into the presence of the body a strange scene occurred; the old lady fell on the corpse, kissed and addressed it by every term of loving endearment, couched in the language of a bygone generation. He was her only love; she waited for him during her long life; she knew that he had not forsaken her. The old woman and the young man had been betrothed sixty years before. The lover had disappeared mysteriously, and she had kept faithful during that long interval. Time had stood still with the dead man, but had left its mark on the living woman. The miners who were present were a rough set, but very gently and with tearful eyes they removed the old lady to her house, and the same night her faithful spirit rejoined that of her long-lost lover."—*Church Union.*

---

The Scotchman and the Mineralogist.—"Yon man gave me his bag to carry by a short cut across the hills to his inn, while he took the other road. Eh! it was dreadful heavy, and when I got out of his sight, I determined to see what was in it, for I wondered at the unco weight of the thing; and man, it's no use for you to guess what was in that bag, for you'd ne'er find out. It was stones." "And did you carry it?" "Carry it, man, did you think I was as mad as himself? Nae! nae! I emptied them all out, but I filled the bag again from the pile near the house, and gave him good measure for his money."

